# Robust Multiple Regression

**DOI:** 10.3390/e23010088

**Published:** 2021-01-09

**Authors:** David W. Scott, Zhipeng Wang

**Affiliations:** 1 Department of Statistics, Rice University, MS-138, 6100 Main Street, Houston, TX 77005, USA; 2 Apple Corporation, Cupertino, CA 95014, USA; zhipeng.wang@alumni.rice.edu

**Keywords:** minimum distance estimation, maximum likelihood estimation, influence functions

## Abstract

As modern data analysis pushes the boundaries of classical statistics, it is timely to reexamine alternate approaches to dealing with outliers in multiple regression. As sample sizes and the number of predictors increase, interactive methodology becomes less effective. Likewise, with limited understanding of the underlying contamination process, diagnostics are likely to fail as well. In this article, we advocate for a non-likelihood procedure that attempts to quantify the fraction of bad data as a part of the estimation step. These ideas also allow for the selection of important predictors under some assumptions. As there are many robust algorithms available, running several and looking for interesting differences is a sensible strategy for understanding the nature of the outliers.

## 1. Introduction

We examine how to approach bad data in the classical multiple regression setting. We are given a section of *n* vectors, $\left\{\left(x_{i},y_{i}\right),i=1,2,\ldots ,n\right\}$. We have *p* predictors; hence, $x_{i}\in {ℜ}^{p}$. The random variable model we consider is $Y_{i}=X_{i}^{t}\beta +\epsilon_{i}$ where $\epsilon_{i}$ represents the (random) unexplained portion of the response. In vector form we have

Y=Xβ+ϵ,

where Y is the n×1 vector of responses. X is the n×p matrix whose *n* rows contain the predictor vectors, and ϵ is the vector of random errors. Minimizing the sum of squared errors leads to the well-known formula

(1) $$\hat{\beta }={\left(X^{t}X\right)}^{-1}X^{t}Y\;.$$

Since $\hat{\beta }$ is a linear combination of the responses, any outliers will result in corresponding influence in the parameter estimates. Alternatively, outliers in the predictor vectors can exert a strong influence on the estimated parameter vector. With modern gigabit datasets, both outliers may be expected. Outliers in the predictor space may or may not be viewed as errors. In either case, they may result in high leverage, as any prediction errors there that are very large would result in a large fraction of the SSE; thus, we would expect $\hat{\beta }$ to pay attention and try to rotate to minimize that effect. In practice, it is more common to assume the features are measured accurately and without error and to focus on outliers in the response space. We will adopt this framework initially.

## 2. Strategies for Handling Outliers in the Response Space

Denote the multivariate normal PDF by ϕ(x|μ,Σ). Although it is not required, if we assume the distribution of the error vector ϵ is multivariate normal with zero mean and covariance matrix $\Sigma =\sigma_{\epsilon }^{2}I_{p}$, maximizing the likelihood

(2) $$\begin{array}{cc} \prod_{i=1}^{n}\;\phi \left(\epsilon_{i}|\beta ,\sigma_{\epsilon }^{2}I_{p}\right) & =\prod_{i=1}^{n}\;\frac{1}{\sqrt{2\pi {\sigma_{\epsilon }}^{2}}}\exp\left(-\epsilon_{i}^{2}/2{\sigma_{\epsilon }}^{2}\right)\\ \; & =\prod_{i=1}^{n}\;\frac{1}{\sqrt{2\pi {\sigma_{\epsilon }}^{2}}}\exp\left(-{\left(y_{i}-x_{i}^{t}\beta \right)}^{2}/2{\sigma_{\epsilon }}^{2}\right) \end{array}$$

may be shown to be equivalent to minimizing the residual sum of squares

(3) $$\begin{array}{cc} \sum_{i=1}^{n}{\left(y_{i}-{\hat{y}}_{i}\right)}^{2} & =\sum_{i=1}^{n}{\left(y_{i}-x_{i}^{t}\beta \right)}^{2}\\ \; & ={\left(Y-X\beta \right)}^{t}\left(Y-X\beta \right)\;, \end{array}$$

over β, leading to the least squares estimator given in Equation (1), where ${\hat{y}}_{i}=x_{i}^{t}\hat{\beta }$ is the predicted response. Again we remark that the least squares criterion in Equation (3) is often invoked without assuming the errors are independent and normally distributed.

Robust estimation for the parameters of the normal distribution as in Equation (2) is a well-studied topic. In particular, the likelihood is modified so as to avoid the use of the non-robust squared errors found in Equation (3). For example, $\epsilon_{i}^{2}$ may be modified to be bounded from above, or may even take a more extreme modification to have redescending shape (to zero); see [1,2,3]. Either approach requires the specification of meta-parameters that explicitly control the shape of the resulting influence function. Typically, this is done by an iterative process where the residuals are computed and a robust estimate of their scale is obtained. For example, the median of the absolute median residuals.

As an alternative, we advocate making an assumption about the explicit shape of the residuals, for example, $\epsilon ∼N(0,{\sigma_{\epsilon }}^{2})$. With such an assumption, it is possible to replace likelihood and influence function approaches with a minimum distance criterion. As we shall show, the advantage of doing so is that an explicit estimate of the fraction of contaminated data may be obtained. In the next section, we briefly describe this approach and the estimation equations.

## 3. Minimum Distance Estimation

We follow the derivation of the L2E algorithm described by Scott [4]. Suppose we have a random sample $\left\{x_{i},i=1,2,\ldots ,n\right\}$ from an unknown density function g(x), which we propose to model with the parametric density f(x|θ). Either *x* or θ may be multivariate in the following. Then as an alternative to evaluating potential parameter values of θ with respect to the likelihood, we consider instead estimates of how close the two densities are in the integrated squared or $L_{2}$ sense:

(4) $$\begin{array}{cc} \hat{\theta } & =\arg\min_{\theta }\;\hat{\int }{\left(f(x|\theta )-g(x)\right)}^{2}dx \end{array}$$

(5) $$\begin{array}{cc} \; & =\arg\min_{\theta }\left[\;\hat{\int }f{\left(x|\theta \right)}^{2}dx-\hat{\int }2f\left(x|\theta \right)g\left(x\right)dx+\hat{\int }g{\left(x\right)}^{2}dx\right] \end{array}$$

(6) $$\begin{array}{cc} \; & =\arg\min_{\theta }\left[\;\int f{\left(x|\theta \right)}^{2}dx-2\hat{E}f\left(X|\theta \right)\right] \end{array}$$

(7) $$\begin{array}{cc} \; & =\arg\min_{\theta }\left[\;\int f{\left(x|\theta \right)}^{2}dx-\frac{2}{n}\sum_{i=1}^{n}f\left(x_{i}|\theta \right)\right]\;. \end{array}$$

Notes: In Equation (4), the hat on the integral sign indicates we are seeking a data-based estimator for that integral; in Equation (5), we have simply expanded the integrand into three individual integrals, the first of which can be calculated explicitly for any posited value of θ and need not be estimated; in Equation (6), we have omitted the hat on the first integral and eliminated entirely the third integral since it is a constant with respect to θ, and we have observed that the middle integral is (by definition) the expectation of our density model at a random point X∼g(x); and finally, in Equation (7), we have substituted an unbiased estimate of that expectation. Note that the quantity in brackets in Equation (7) is fully data-based, assuming the first integral exists for all values of θ. Scott calls the resulting estimator L2E as it minimizes an $L_{2}$ criterion.

We illustrate this estimator with the 2-parameter $N(\mu ,\sigma^{2})$ model. Then the criterion in Equation (7) becomes

(8) $$\begin{array}{cc} \left(\hat{\mu },\hat{\sigma }\right) & =\arg\min_{\left(\mu ,\sigma \right)}\;\left[\frac{1}{2\sqrt{\pi }\sigma }-\frac{2}{n}\sum_{i=1}^{n}\phi \left(x_{i}|\mu ,\sigma^{2}\right)\right]\;. \end{array}$$

**Figure 1:**
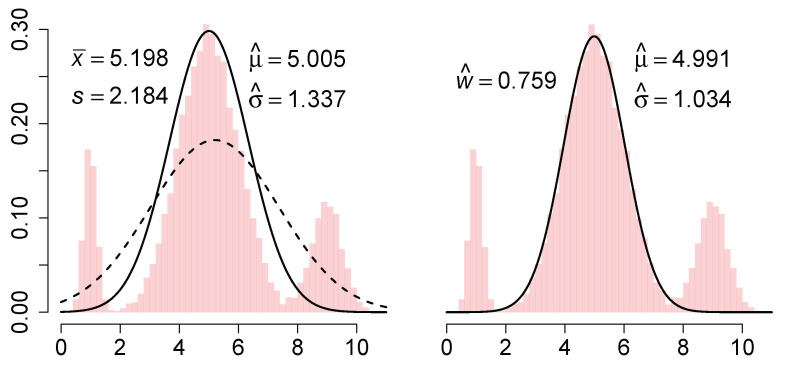
(**Left**) MLE and L2E estimates together with a histogram; (**Right**) partial L2E estimate.

We illustrate this estimator on a sample of $10^{4}$ points from the normal mixture $0.10N\left(1,0.2^{2}\right)+0.75N\left(5,1\right)+0.15N\left(9,0.5^{2}\right)$. The L2E and MLE curves are shown in the left frame of Figure 1.

A careful examination of the L2E derivation in Equation (4) shows that we crucially used the fact that g(x) was a density function, but nowhere did we require the model f(x|θ) to also be a bona fide density function. Scott proposed fitting a partial mixture model, namely

$$f\left(x|\theta \right)=w\cdot \phi \left(x|\mu ,\sigma^{2}\right)\;,$$

which he called a partial density component. (Here, the L2E criterion could be applied to a full 3-component normal mixture density.) When applied to the previous data, the fitted curve is shown in the right frame of Figure 1.

We discuss these 3 estimators briefly. The MLE is simply $\left(\bar{x},s\right)$, and the nonrobustness of both parameters is clearly illustrated. Next, the L2E estimate of the mean is clearly robust, but the scale estimate is also inflated compared to the true value σ=1. After reflection, this is the result of the fitted model having an area equal to 1. The closest normal curve is close to the central portion of the mixture, but with standard deviation inflated by a third. Note that the fitted curve completely ignores the outer mixture components. However, when the 3-parameter partial density component model is fitted, $\hat{w}=0.759$, which suggests that some 24% of the data are not captured by the minimum distance fit. Thus the estimation step itself conveys important information about the adequacy of the fit. By way of contrast, a graphical diagnosis of the MLE fit such as a *q*–*q* plot would show the fit is also inadequate, but give no explicit guidance as to how much data are outliers and what the correct parameters might be. Note that the parameter estimates of the mean and standard deviation by partial L2E are both robust, although the estimate of σ is inflated by 3%, reflecting some overlap of the third mixture component with the central component. Thus, we should not assume $\hat{w}$ is an unbiased estimate of the fraction of “good data”, but rather an upper bound on it.

With the insight gained by this example, we shift now to the problem at hand, namely, multiple regression. We will use the partial L2E formulation in order to gain insight into the portion of data not adequately modeled by the linear model.

## 4. Minimum Distance Estimation for Multiple Regression

If we are willing to make the (perhaps rather strong but explicit) assumption that the error random variables follow a normal distribution, the appropriate model is

$$\epsilon ∼N(0,{\sigma_{\epsilon }}^{2})\;.$$

Given initial estimates for β, $\sigma_{\epsilon }$, and *w*, we use any nonlinear optimization routine (for example, *nlminb* in the R language) to minimize Equation (7)

(9) $$\frac{w^{2}}{2\sqrt{\pi }\sigma_{\epsilon }}-\frac{2w}{n}\sum_{i=1}^{n}\phi \left(y_{i}-x_{i}^{t}\beta |0,{\sigma_{\epsilon }}^{2}\right)$$

over the p+2 parameters $\left(\beta ,\sigma_{\epsilon },w\right)$. In practice, the intercept may be coded as another parameter, or a column of 1s may be included in the design matrix, X. Notice that the residuals are assumed to be normal (at least partially) and centered at 0. It is convenient to use the least-squares estimates to initialize the L2E algorithm. In some cases, there may be more than one solution to Equation (9), especially if using the partial component model. In every case, the fitted value of *w* should offer clear guidance.

## 5. Examples

### 5.1. Hertzsprung–Russell Diagram CYG OB1 Data

**Figure 2:**
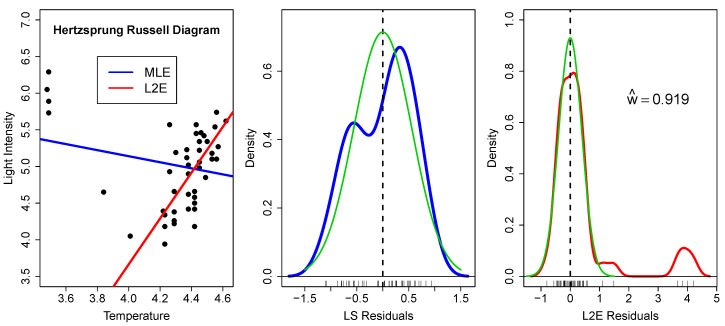
(**Left**) MLE (blue) and L2E (red) regression estimates for the Hertzsprung–Russell data; (**Middle**) kernel (blue) and normal (green) densities of the least squares residuals; and (**Right**) kernel (red) and normal (green) densities of the L2E residuals. See text.

These data (n=47) are well-studied due to the strong influence of the four very bright giant stars observed at low temperatures [5]; see Figure 2. In fact, the slope of the least-squares line in the left frame has the wrong sign.

In the middle frame, we examine the residuals from the least-squares fit. The residuals are shown along the *x*-axis, together with a kernel density estimate (blue), which has a bimodal shape [6]. The green curve shows the presumed normal fit $N(0,{\hat{\sigma }}_{\epsilon }^{2})$, where ${\hat{\sigma }}_{\epsilon }=0.558$. Since this is just a bivariate case, it is easy to see that the bimodal shape of the residuals does not convey the correct size of the population of outliers. In higher dimensions, such inference about the nature and quantity of outliers only becomes more difficult.

In the right frame, we examine the residuals from the L2E fit. We begin by noting that the fraction of “good data” is around 92%, indicating 3.8 outliers. The kernel density estimate of the residuals is shown in red. The fitted normal curve to the residuals is the partial normal component given by

$$0.919\cdot N(0,0.394^{2})$$

and is shown again in green. The estimated L2E standard deviation is 41% smaller than the least-squares estimate. Examining the residuals closely, there are a possible two more stars with residual values 1.09 and 1.49 that may bear closer scrutiny. Finally, the assumption of a normal shape for the residuals seems warranted by the close agreement of the red and green curves around the origin in this figure.

### 5.2. Boston Housing Data

This dataset was first analyzed by economists who were interested in the affect that air pollution (nitrous oxide) had on median housing prices per census track [7]. A description of the data may be found at https://www.cs.toronto.edu/~delve/data/boston/bostonDetail.html.

We begin by fitting the full least-squares and L2E multiple regression models with p=13 predictors to the median housing price for the 506 census tracks. All 14 variables were standardized; see Table 1. Thus we know the intercept for the least-squares model will be zero. All of the LS coefficients were significant except for INDUS and AGE. L2E puts more weight on AGE and RM and less on NOX, RAD, and LSTAT compared to least-squares.

**Figure 3:**
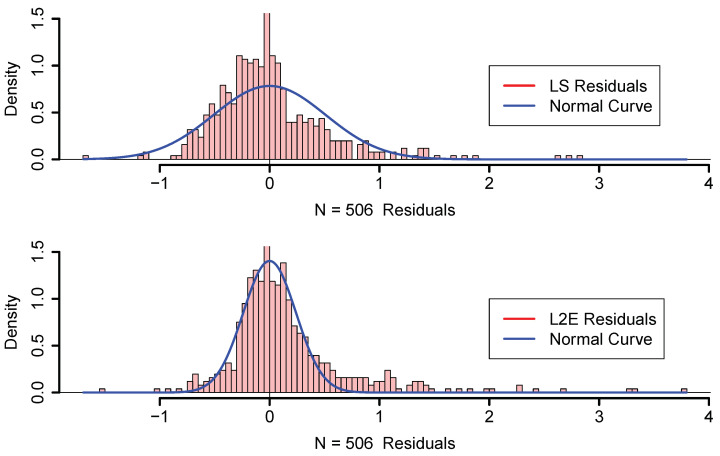
LS and L2E residual analysis; see text.

In Figure 3, we display histograms of the residuals as well as a normal curve with mean zero and the estimated standard deviation of the residuals. The estimated value of $\sigma_{\epsilon }$ is 0.509 and $R^{2}=0.74$ for LS; however, ${\hat{\sigma }}_{\epsilon }$ is only 0.240 for L2E, with $\hat{w}=0.845$. Examining the curves in Figure 3, we see that the least-squares model tends to over-estimate the median housing value. Our interpretation of the L2E result is that the simple multiple linear regression model only provides an adequate fit to at most 84.5% of the data. (This interpretation relies critically on the correctness of the proper shape of the residuals following the normal distribution.) In particular, the L2E model is saying that very accurate predictions of the most expensive median housing census tracks are not possible with these 13 predictors.

**Figure 4:**
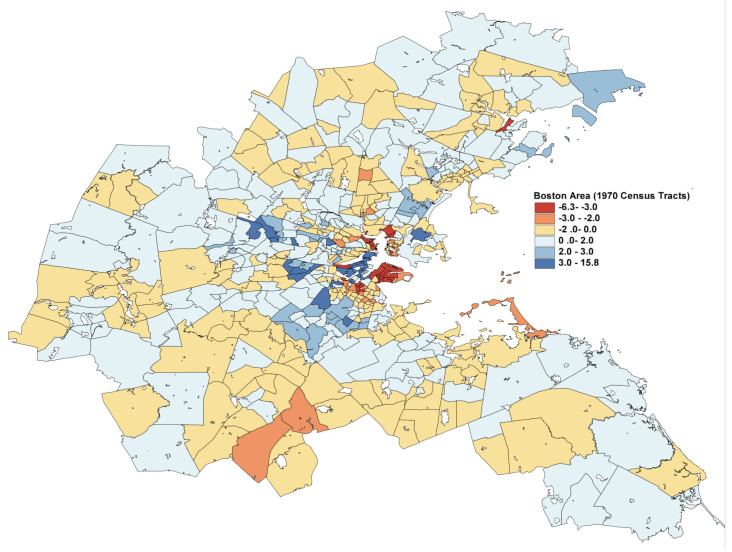
Full map of the L2E residuals in the Boston region; see text.

**Figure 5:**
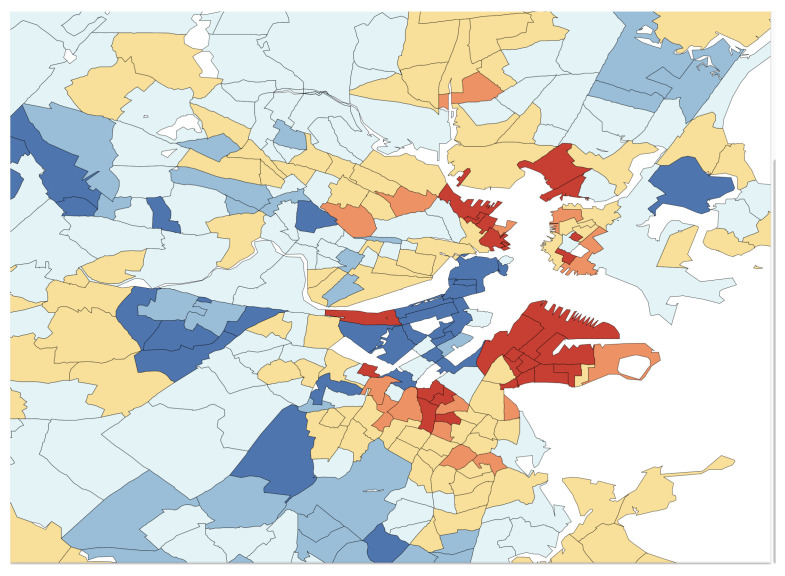
Blow-up of central Boston region residuals.

In Figure 4, the L2E residuals (in standardized units) are displayed for the 506 census tracks. The dark blue and dark red shaded tracks are more than 3 standard units from their predictions. The expanded scale shown in Figure 5 shows that the largest residuals (outliers) are in the central Boston to Cambridge region. Similar maps of the LS residuals show much less structure, as is apparent from the top histogram in Figure 3.

**Figure 6:**
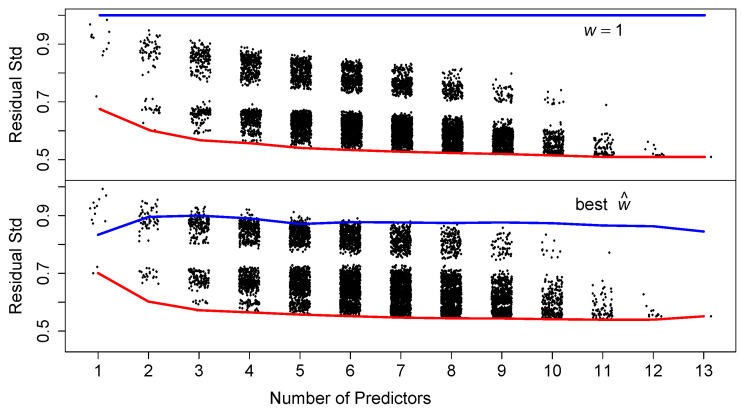
Fitting all possible subsets of predictors for the median housing values ($2^{13}-1=8191$). The red lines connect the best model for each number of predictors. The blue lines connect the best *w* for that best model. Of course w=1 for all least-squares models. See text.

Next, we briefly examine whether subsets of the predictors are sufficient for prediction. In Figure 6, we display the residual standard deviation for all 8191 such models. Apparently, as few as 5 variables provide as good a prediction as the full model above. In the bottom two rows of Table 1 we tabulate the variables that entered into the best 100 models as the number of variables range from 5 to 8. The variables RM, LSTAT, PTRATIO, and DIS appear in almost all of those 400 models. The additional three variables ZN, B, and CHAS appear at least half the time. However, the L2E fits for these models have standard errors often 50% larger than the full model. Variable selection remains a noisy process, although this simple counting procedure can prove informative.

### 5.3. Superconductivity Data

This dataset was analyzed in 2018 in a published manuscript [8] to predict the superconductivity critical temperature using the features extracted from the superconductor’s chemical formula. A description of the dataset may be found at https://archive.ics.uci.edu/ml/datasets/Superconductivty+Data#.

**Figure 7:**
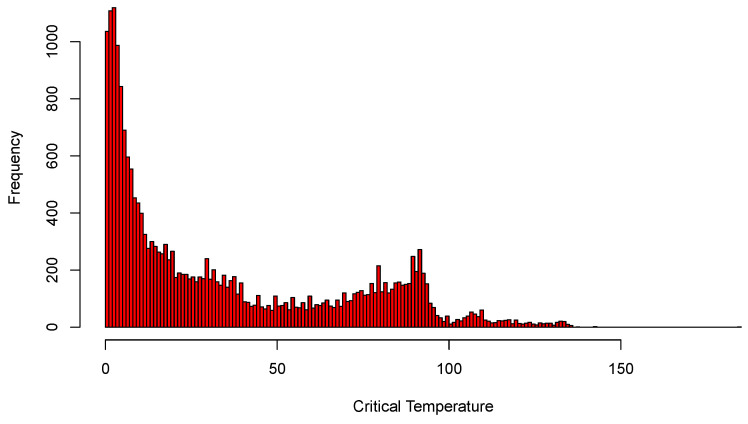
Histograms of the critical temperatures; see text.

**Figure 8:**
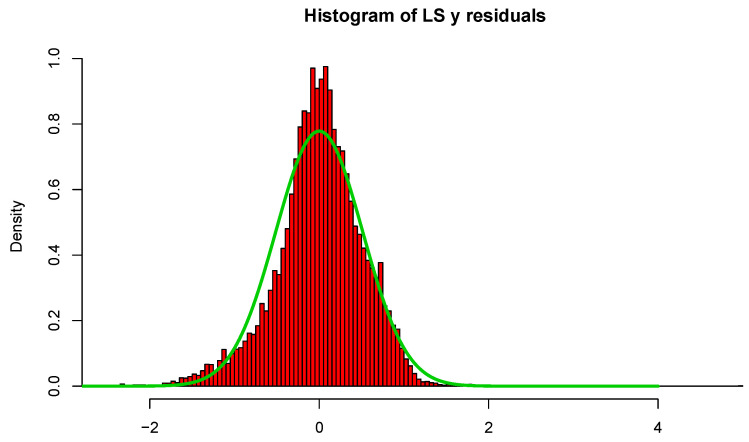
Histogram and normal curve for LS residual; see text.

As for the other examples, we begin by fitting the full least-squares and L2E multiple regression models with p=81 predictors to the critical temperature for the 21,263 superconductors. All 82 variables were standardized; in Figure 7, we display histograms of the critical temperatures of the 21,263 superconductors. The data clearly manifest two “major clusters” and one “minor cluster”. We also display histograms of the least-squares regression residuals as well as a normal curve with mean zero and the estimated standard deviation of the residuals. When we examine the histogram and curves in Figure 8, we see that the least-squares model overall does a reasonable job, while possessing larger deviation.

**Figure 9:**
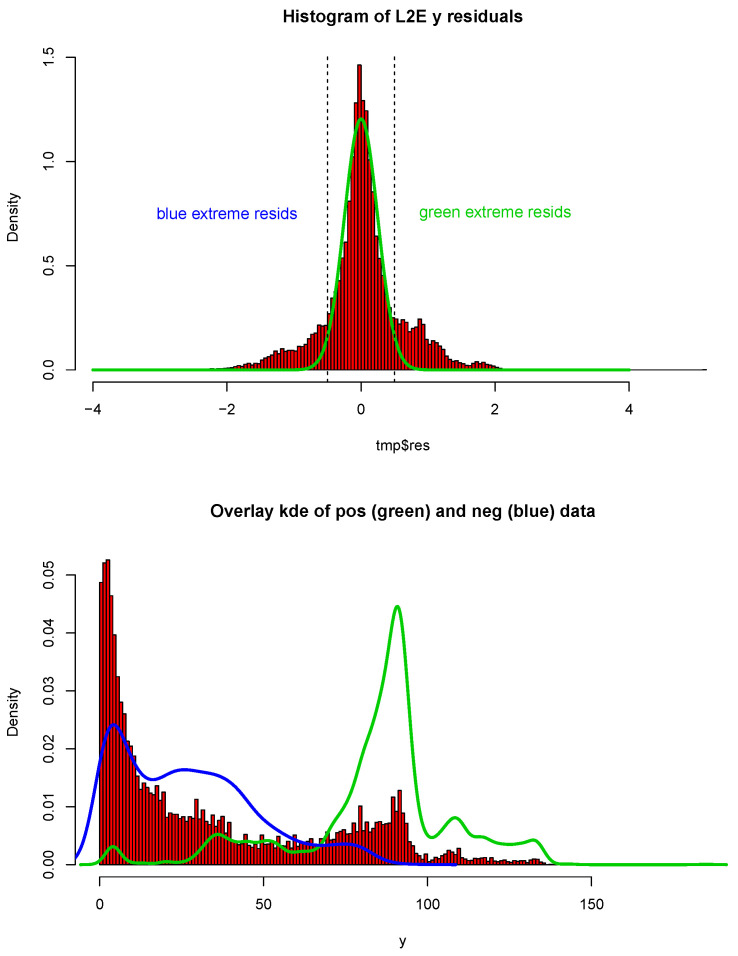
Histogram and fitting kernel density estimation curves for L2E residuals; see text.

We showcase the histograms and curves for L2E regression residuals, as well as the fitting curves in Figure 9. We plotted the blue curve with the negative residuals and the green curve with positive residuals. Our interpretation of the L2E result is that the points with positive and negative residuals from the L2E regression fit the two major clusters of the critical temperature very well. In particular, the L2E model yields a narrower distribution of residuals, and the fitting explains the bi-modal distribution of the critical temperatures. On a practical note, the same L2E values (to five significant digits) were obtained starting with the LS parameters or a vector of zeros, for any initial choice of *w*.

## 6. Discussion

Maximum likelihood or entropy or Kullbach–Liebler estimators are examples of divergence criteria rather than being distance-based. It is well-known these are not robust in their native form. Donoho and Liu argued that all minimum distance estimators are inherently robust [9]. Other minimum distance criteria (L1 or Hellinger, e.g.) exist with some properties superior to L2E such as being dimensionless. However, none are fully data-based and unbiased. Often a kernel density estimate is placed in the role of g(x), which introduces an auxiliary parameter that is problematic to calibrate. Furthermore, numerical integration is almost always necessary. Numerical optimization of a criterion involving numerical integration severely limits the number of parameters and the dimension that can be considered.

The L2E approach with multivariate normal mixture models benefits greatly from the following closed form integral:

$$\int_{{ℜ}^{p}}\;\phi \left(x|\mu_{1},\Sigma_{1}\right)\;\phi \left(x|\mu_{2},\Sigma_{2}\right)\;dx=\phi \left(0|\mu_{1}-\mu_{2},\Sigma_{1}+\Sigma_{2}\right)\;,$$

whose proof follows from the Fourier transform of normal convolutions; see the appendix of Wand and Jones [10]. Thus the robust multiple regression problem could be approached by fitting the parameter vector (μ,Σ,w) to the random variable vector (x,y) and then computing the conditional expectation. In two dimensions, the number of parameters is 2+3+1 compared to the multiple regression parameter vector $\left(\beta ,\sigma_{\epsilon },w\right)$, which has 2 + 1 + 1 parameters (including the intercept). The advantage is much greater as *p* increases, as the full covariance matrix requires p(p+1)/2 parameters alone.

**Figure 10:**
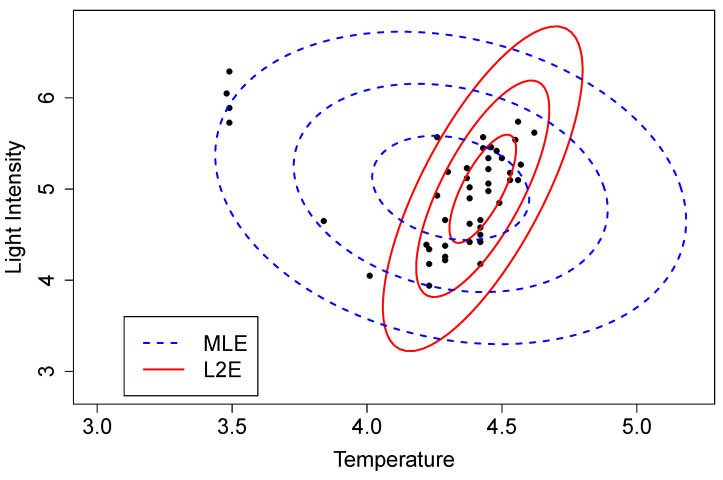
MLE (blue) and L2E (red) bivariate normal estimates for the Hertzsprung–Russell data.

To illustrate this approach, we computed the MLE and L2E parameters estimates for the Hertzsprung–Russell data [11]. The solutions are depicted in Figure 10 by three level sets corresponding to 1-, 2-, and 3-σ contours. These data are not perfectly modeled by the bivariate normal PDF; however, the direct regression solutions shown in the left frame of Figure 2 are immediately evident. The estimate of $\hat{w}$ here was 0.937, which is slightly larger than the estimate shown in the right frame of Figure 2.

If the full correlation structure is of interest, then the extra work required to robustly estimate the parameters may be warranted. For $x\in {ℜ}^{p}$, this requires estimation of p+p(p+1)/2+1 or (p+2)(p+1)/2 parameters. In ${ℜ}^{10}$ this means estimating 66 parameters, which is on the edge of optimization feasibility currently. Many simulated bivariate examples of partial mixture fits with 1–3 normal components are given in Scott [11]. When the number of fitted components is less than the true number, initialization can result in alternative solutions. Some correctly isolate components, others combine them in interesting ways. Software to fit such mixtures and multiple regression models may be found at http://www.stat.rice.edu/~scottdw/ under the *Download software and papers* tab.

We have not focused on the theoretical properties of L2E in this article. However, given the simple summation form of the L2E criterion in Equation (7), the asymptotic normality of the estimated parameters may be shown. Such general results are to be found in Basu, et al. [12], for example. Regularization of L2E regression, such as the $L_{1}$ penalty in LASSO, has been considered by Ma, et al. [13]. LASSO can aid in the selection of variables in a regression setting.

## 7. Conclusions

The ubiquitousness of massive datasets has only increased the need for robust methods. In this article, we advocate application of numerous robust procedures, including L2E, in order to find similarities and differences among their results. Many robust procedures focus on high-breakdown as a figure of merit; however, even those algorithms may falter in the regression setting; see Hawkins and Olive [14]. Manual inspection of such high-dimensional data is not feasible. Similarly, graphical tools for inspection of residuals also are of limited utility; however, see Olive [15] for a specific idea for multivariate regression. The partial L2E procedure described in this article puts the burden of interpretation where it can more reasonably be expected to succeed, namely, in the estimation phase. Points tentatively tagged as outliers may still be inspected in aggregate for underlying cause. Such points may have residuals greater than some multiple of the estimated residual standard deviation, $\hat{\sigma_{\epsilon }}$, or simply be the largest $100(1-\hat{w})\%$ of the residuals in magnitude. In either case, the understanding of the data is much greater than least-squares in the high-dimensional case.

## Figures and Tables

**Table 1:** The multiple regression parameter estimates for LS and L2E are given in the first two rows. The variable importance counts are given in the last two rows; see text.

	Int	CRIM	ZN	INDUS	CHAS	NOX	RM	AGE	DIS	RAD	TAX	PTRATIO	B	LSTAT
LS	0	−0.101	0.118	0.015	0.074	−0.224	0.291	0.002	−0.338	0.290	−0.226	−0.224	0.092	−0.407
L2E	−0.155	−0.140	0.078	0.015	0.048	−0.061	0.400	−0.135	−0.177	0.105	−0.135	−0.135	0.173	−0.180
LS		123	121	112	158	134	396	110	270	128	137	334	155	396
L2E		135	155	133	157	133	396	63	269	122	144	305	166	396
